# Mesenchymal stem cells and derived extracellular vesicles in major respiratory diseases: from multifaceted molecular mechanisms to clinical perspectives

**DOI:** 10.3389/fcell.2026.1839015

**Published:** 2026-06-01

**Authors:** Ang Li, Xiaomin Wang, Jiaqi Wu, Guojie Zhao, Na Li, Zhao Wang, Yaqin Li, Yanfu Wang

**Affiliations:** 1 Department of General Medicine, The First Affiliated Hospital of Dalian Medical University, Dalian, Liaoning, China; 2 Dalian Medical University, Dalian, Liaoning, China

**Keywords:** acute respiratory distress syndrome, asthma, bronchopulmonary dysplasia, lung cancer, mesenchymal stem cells, pulmonary arterial hypertension, pulmonary fibrosis

## Abstract

Mesenchymal stem cells (MSCs), a type of pluripotent stem cells, exhibit remarkable therapeutic properties in the treatment of various diseases. Recent findings on the development of respiratory illnesses indicate that local MSCs might transform into myofibroblasts, thereby resulting in the emergence of respiratory disorders. Nonetheless, both preclinical and clinical studies on autologous or allogeneic MSC infusion reveal that MSCs can mitigate the onset of respiratory disease by controlling inflammation, restoring impaired tissues, modifying the ECM, and regulating stress-induced cell death after implantation. Furthermore, a variety of animal models have been created to explore the underlying mechanisms of various respiratory illnesses and assess the early-stage efficacy of MSC treatment in these conditions. In summary, the article examines the divergent functions of MSCs in respiratory illnesses and summarizes current perspectives on their therapeutic mechanisms in these diseases.

## 1 Introduction

### 1.1 History of MSCs

Mesenchymal stromal/stem cells (MSCs) are multipotent adult stromal cells with immunomodulatory, trophic, and tissue-reparative properties. Increasing evidence indicates that MSCs and MSC-derived extracellular vesicles (MSC-EVs) participate in the progression and repair of major respiratory diseases by regulating inflammatory responses, epithelial and endothelial barrier integrity, extracellular matrix remodeling, oxidative stress, and intercellular communication. However, the biological effects of MSCs are context dependent: endogenous or disease-associated stromal cell populations may contribute to fibrotic remodeling, whereas exogenously administered MSCs or MSC-EVs may exert protective effects in selected preclinical and early clinical settings. This review summarizes current evidence on the roles of MSCs and MSC-EVs in acute respiratory distress syndrome, bronchopulmonary dysplasia, pulmonary arterial hypertension, pulmonary fibrosis, lung cancer, and asthma. Particular attention is given to disease pathobiology, proposed mechanisms of action, the level of available evidence, and key translational constraints, including cell identity, product quality, EV characterization, dosing metrics, storage, and safety (Yan et al., 2023; Miclau et al., 2023; Wu F. et al., 2023). Overall, MSC-based approaches remain promising but require standardized characterization and rigorously designed clinical trials before broad clinical application.

### 1.2 Markers of MSCs

Primarily, MSC biomarkers such as CD73, CD90, and CD105 play a crucial role in determining their purity and properties (Xu et al., 2022; Azhdari et al., 2022). Furthermore, the absence of hematopoietic markers CD34 and CD45 in MSCs aids in differentiating them from other cell types (Zargar et al., 2022). While MSCs originating from varied locations like adipose tissue and umbilical cord exhibit diverse surface marker expressions, the shared nature of these core markers lays the groundwork for their investigative work (Zargar et al., 2022). Comprehensive studies have revealed that MSCs possess several biological properties relevant to regenerative medicine and cell therapy, particularly their capacity for self-renewal, immunomodulation, paracrine signaling, and multipotent differentiation toward mesodermal lineages under appropriate *in vitro* conditions (Guo et al., 2023).

### 1.3 MSC identity, product quality, and EV nomenclature

MSC-EVs are heterogeneous membrane-bound particles released by MSCs and include small EVs, medium/large EVs, and vesicles sometimes referred to as exosomes when their endosomal origin is experimentally supported. Because the term ‘exosome’ is often used inconsistently, we use ‘MSC-EVs’ as the general term unless the biogenesis or isolation method justifies a more specific nomenclature. EV characterization should include, where possible, particle size and concentration analysis, morphology assessment, detection of EV-associated markers such as CD9, CD63, CD81, TSG101, or ALIX, and evaluation of negative or contaminating markers. Source heterogeneity, isolation procedures, dose metrics, storage conditions, freeze-thaw cycles, and normalization strategies can substantially affect EV cargo composition and functional readouts (Abbaszadeh et al., 2022; Saleh et al., 2022; Zheng et al., 2023). These issues are particularly important when comparing isolated MSC-EVs with the broader MSC secretome, which also contains soluble cytokines, growth factors, lipids, metabolites, and extracellular matrix-associated components.

### 1.4 Overview of the impact of MSCs on disease progression

Presently, MSCs are crucial in the development of a range of diseases. Initially, their impact on ARDS treatment is notable, as they secrete anti-inflammatory factors and aid in tissue healing (Curley et al., 2024; Liang et al., 2023). Furthermore, MSCs are capable of modulating immune reactions and exhibit superior regulatory capacities in autoimmune disorders and transplant settings (Aghayan et al., 2022; Batchinsky et al., 2023). Furthermore, the healing impact of MSCs on heart conditions, liver damage, and other diseases has also been validated through clinical trials (Morrison et al., 2017; Wang et al., 2023).

Generally, MSCs, being multifunctional cells, hold extensive potential for clinical use; however, additional studies are required to investigate their underlying mechanisms and refine treatment strategies. The focus of this paper is to highlight the functions of MSCs in regulating respiratory illnesses, with the goal of summarizing current research advances in this area and identifying future research directions.

## 2 The impact of MSCs on lung diseases

### 2.1 The role and impact of MSCs on ARDS

What is the potential trajectory of cell therapy development for acute respiratory distress syndrome (ARDS) in the future? Presently, it is imperative to focus on this specific domain. Relevant studies suggest that the management of ARDS is predominantly based on supportive care, including mechanical ventilation and fluid management. Unfortunately, the lack of effective therapeutic interventions results in a poor prognosis for the majority of ARDS patients. Survivors often endure persistent pulmonary dysfunction and cognitive impairment, which severely affect their quality of life. Emerging research highlights that the application of MSCs and their exosomes has opened a promising and expansive field for addressing the challenges encountered by patients with ARDS (Liu P. et al., 2024). Current studies indicate that MSCs and their exosomes exhibit a range of beneficial properties, notably in immune modulation, inflammation reduction, promotion of cellular repair and regeneration, and inhibition of cell death (Su et al., 2023).

#### 2.1.1 Immune regulatory effect

Studies indicate that MSCs modulate the immune response by secreting anti-inflammatory cytokines such as IL-10 and TGF-β, thereby inhibiting the activation and infiltration of inflammatory cells, including macrophages and neutrophils. This immunomodulatory effect contributes to the reduction of lung inflammation, helping to alleviate the tissue damage associated with ARDS (Chen et al., 2023; Dutra Silva et al., 2021). MSCs can also influence macrophage polarization by promoting a shift from the pro-inflammatory M1 phenotype to the anti-inflammatory M2 phenotype, which subsequently reduces the production of inflammatory mediators such as TNF-α and IL-6. This phenotypic transition plays a crucial role in suppressing both local pulmonary and systemic inflammation (Matthay et al., 2019).

Furthermore, human umbilical cord-derived MSCs secrete Stanniocalcin-1 (STC-1), which enhances IL-10 expression in alveolar macrophages through activation of the PI3K/AKT/mTOR signaling pathway (Fatima et al., 2024). In models of Klebsiella-induced ARDS, placental MSCs have been shown to promote M2 polarization of alveolar macrophages rather than M1 polarization via an IL-12-dependent mechanism. MSC-derived exosomes have also demonstrated protective effects by preventing pyroptosis of alveolar macrophages, thereby reducing acute lung injury (Edström et al., 2023). Additionally, EVs released by MSCs exert therapeutic effects in ARDS by reprogramming macrophage responses through the miR-181a/STAT5/SOCS1 signaling axis, further enhancing their anti-inflammatory potential (Iglesias et al., 2021).

#### 2.1.2 Cell repair and regeneration

Studies have shown that MSCs promote the proliferation and regeneration of alveolar epithelial cells by secreting various growth factors and cytokines, such as vascular endothelial growth factor (VEGF), hepatocyte growth factor (HGF), and others. The repair of damaged lung tissue is crucial and can significantly improve pulmonary function (Zhang and Slutsky, 2023; Bowdish et al., 2023). In addition to supporting alveolar epithelial cell growth, MSCs also stimulate angiogenesis, enhance pulmonary blood flow, facilitate oxygen exchange, and aid in carbon dioxide elimination—processes essential for the restoration of functional lung tissue (Masterson et al., 2021). Furthermore, research suggests that elevated levels of Wnt5a may enhance the protective effects of MSCs against lipopolysaccharide-induced endothelial cell injury by activating the PI3K/AKT signaling pathway (Lv et al., 2020).

#### 2.1.3 Oxygenation improvement

MSCs hold significant potential to enhance oxygenation capacity by improving the function of the alveolar-capillary interface. This is achieved through multiple mechanisms, including the promotion of pulmonary microcirculation, reduction of pulmonary edema, and enhancement of alveolar gas exchange efficiency (Lopes-Pacheco et al., 2020; Chen F. et al., 2024). Notably, miR-202-5p, a microRNA enriched in extracellular vesicles derived from bone marrow MSCs, has been shown to inhibit pyroptosis by targeting CMPK2, thereby attenuating lung ischemia-reperfusion injury. Intriguingly, administration of MSCs has been demonstrated to improve systemic oxygen utilization, alleviate hypoxemia, and enhance overall physiological status in affected individuals. Furthermore, upregulation of FoxM1 has been found to enhance the therapeutic efficacy of bone marrow MSCs in ARDS by improving their oxygenation-promoting effects. These findings are supported by multiple independent studies (Valiukevičiu et al., 2023; Zhang et al., 2023).

#### 2.1.4 The role of extracellular vesicles

Numerous bibliometric analyses indicate that interventions utilizing MSC-derived exosomes can effectively mitigate the inflammatory response associated with ARDS. MSCs modulate the surrounding microenvironment by secreting EVs, which serve as vehicles for bioactive molecules such as miRNAs and proteins. These extracellular vesicles play a crucial role in repairing damaged cells and enhancing anti-inflammatory capabilities (Lv et al., 2024; Millar et al., 2020).

MSCs release EVs that modulate immune cell functions, promote the polarization of macrophages toward an anti-inflammatory phenotype, and suppress the release of pro-inflammatory mediators, thereby alleviating lung injury (Cyr-Depauw et al., 2024). Studies have shown that endothelial cell-derived EVs regulate the therapeutic effects of MSCs in ARDS through the IDH2/TET signaling pathway. Similarly, MSC-derived EVs have been found to attenuate viral pneumonia-associated inflammation in murine macrophages by inhibiting the NF-κB/NLRP3 inflammatory signaling axis (Abele et al., 2022). Elevated expression of CXCR7 in umbilical cord-derived MSCs enhances their ability to mitigate ARDS-associated pulmonary fibrosis by suppressing the Notch/Jag1 pathway, a process mediated by Wnt/β-catenin signaling. Human placental MSCs and their secreted EVs have demonstrated efficacy in reducing lung damage in murine models of ARDS (Benny et al., 2022). Additionally, MSCs alleviate H9N2-induced acute lung injury by inhibiting caspase-3-mediated apoptosis in alveolar epithelial cells (Moreira et al., 2020). Furthermore, EVs derived from human placental MSCs help restore endothelial barrier integrity by modulating the cytoskeleton via the hsa-miR-148a-3p/ROCK1 signaling pathway (Hazra et al., 2022).

#### 2.1.5 Antioxidant effect

By enhancing the production of antioxidant enzymes such as superoxide dismutase (SOD) and catalase (CAT), MSCs can effectively reduce intracellular reactive oxygen species (ROS) levels. This mechanism helps protect lung cells from oxidative damage and alleviates ARDS-associated lung injury (Klinger and Matthay, 2022). Furthermore, MSCs attenuate lung inflammation and promote cell survival by inhibiting oxidative stress-related signaling pathways, including the NF-κB pathway (Chaudhury et al., 2019).

**FIGURE 1:**
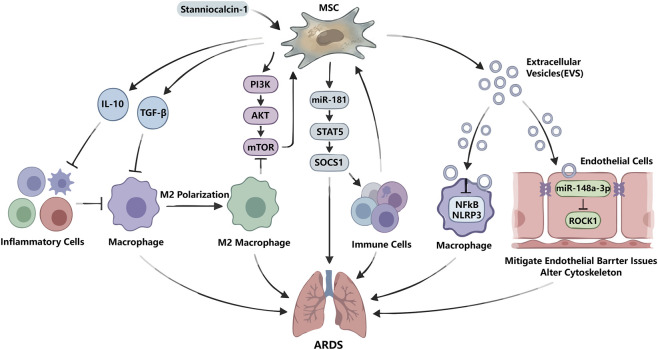
MSCs control the immune response through the release of anti-inflammatory agents IL-10 and TGF - β, preventing the activation and penetration of inflammatory cells. This process aids in diminishing the lungs’ inflammatory reaction. It also has the capability to encourage macrophages to convert into M2, thereby reducing both local and widespread inflammation. Stanniocalcin-1 influences MSCs via the PI3K/AKT/mTOR route. Additionally, the miR-181-STAT5-SOCS1 pathway has the capability to alter immune cells and influence MSCs. Studies have demonstrated that extracellular vesicles can mitigate viral pneumonia in mouse macrophages via the NF kappaB/NLRP3 signaling route. Studies also verify that extracellular vesicles alter the cytoskeleton and mitigate endothelial barrier issues via the miR-148a-3p/ROCK1 route.

Collectively, these studies demonstrate that MSCs exert potent therapeutic effects in ARDS through multiple mechanisms—such as immunomodulation, tissue repair, improvement of oxygenation, exosome-mediated effects, and antioxidant activity. The synergistic interaction of these pathways positions MSCs as a promising therapeutic strategy for ARDS; however, further large-scale clinical trials are needed to fully establish their long-term efficacy and safety (Figure 1).

Although MSCs and MSC-EVs have shown anti-inflammatory and barrier-protective effects in ARDS models, the available clinical evidence remains heterogeneous. ARDS is a syndrome with diverse etiologies, including sepsis, viral pneumonia, trauma, aspiration, and transfusion-related lung injury; therefore, therapeutic responses may vary according to disease trigger, timing of administration, baseline severity, and concomitant organ support. Current studies also differ with respect to cell source, dose, route of delivery, and outcome definitions. Future trials should incorporate mechanistic biomarkers, standardized potency assays, and stratified enrollment strategies to identify patients most likely to benefit.

### 2.2 The role and impact of MSCs on bronchopulmonary dysplasia

During the late stage of lung development, the formation of alveoli and microvessels occurs, playing a vital role in guaranteeing adequate and efficient gas exchange. In premature infants, abnormalities in late lung development manifest as a chronic lung disease known as bronchopulmonary dysplasia. Resident pulmonary interstitial stromal cells in bronchopulmonary dysplasia have been identified using single-cell RNA sequencing (Ng et al., 2020). Analysis of cell communication reveals that the primary drivers of hyperoxia-induced transcriptomic alterations in L-MSCs are inflammatory signals from immune and endothelial cells.

Similar to their role in acute respiratory distress syndrome, MSCs have the capacity to regulate inflammation by secreting anti-inflammatory cytokines such as IL-10, TGF-β, and HGF, which are anti-inflammatory. Such elements contribute to reducing lung inflammation in the BPD mouse model and aid in the regeneration of lung tissue (O'Reilly et al., 2020; Xia et al., 2023). Research indicates that MSCs are capable of modifying macrophage polarization, facilitating their shift to the M2 type (anti-inflammatory type), diminishing inflammatory factor secretion, and consequently diminishing the pro-inflammatory reaction in the lungs (Hao et al., 2022). Immune regulation helps establish a balanced immune microenvironment and promotes lung development.

Beyond modulating immune cells, MSCs promote lung development through the release of growth factors like VEGF, FGF, and TGF-β, thereby supporting the proliferation and differentiation of alveolar epithelial cells. Such growth factors are capable of promoting the development and structural enhancement of alveoli, which in turn improves pulmonary function (Hazra et al., 2022; Klinger and Matthay, 2022). MSCs play a dual role in fostering alveolar growth and the formation of pulmonary blood vessels. Ensuring adequate blood flow is vital for facilitating gas transfer in alveoli and preserving oxygen levels (Dong et al., 2022).

Studies indicate that bronchopulmonary dysplasia frequently coincides with heightened oxidative stress. Research indicates that MSCs can mitigate oxidative damage by elevating the levels of antioxidant enzymes like superoxide dismutase (SOD) and catalase (CAT), thereby reducing intracellular reactive oxygen species (ROS) levels (Chen C. et al., 2024; Zhang Z. et al., 2022). MSCs are capable of enhancing lung cell survival and aiding in healthy lung growth by obstructing apoptotic signaling pathways like p53 and Bax pathways (Caplan, 2016). This protective effect is particularly important in premature infants. Research indicates that MSCs enhance the binding of miR-21 to SKP2 in pulmonary cells, leading to a decrease in Nr2f2 ubiquitination and an increase in Nrf2 attachment to the C/EBP promoter, thereby mitigating hyperoxia-induced lung injury. Hypoxic preconditioning in a rat bronchopulmonary dysplasia model improves both the survival rate and angiogenic capacity of transplanted MSCs via HIF-1 signaling (Caplan, 2016). In cases of lung damage caused by neonatal hypoxia, MSCs diminish the activation of TLR9 mediated by extracellular mitochondrial DNA. Similar studies have also shown that umbilical cord-derived MSCs mitigate hypoxia-induced multi-organ damage via the JAK/STAT pathway (Shokravi et al., 2022). Vesicles outside the cell, originating from MSCs of adipose tissue, mitigate lung damage in newborn rats caused by high oxygen levels by blocking the NF-κB signaling pathway (Yang et al., 2017).

Beyond modulating oxidative stress, it has been demonstrated that MSCs influence bronchopulmonary dysplasia outcomes by enhancing ventilation (Xu et al., 2021). Experimental studies have demonstrated that administering MSCs markedly enhances the respiratory function and oxygenation in mouse models of BPD (Chen et al., 2021). MSCs boost oxygen uptake through the restoration of alveolar architecture, alleviation of pulmonary edema, and enhancement of gas exchange capacity (Yang et al., 2022).

As research progressively intensifies, it becomes critical to investigate the substances playing a pivotal role in the signaling interplay of bronchopulmonary dysplasia cells (Thoreau and Mouthon, 2024). Extracellular vesicles, the primary mediators of intercellular communication, have gained significant attention in this area. Studies indicate that the extracellular vesicles secreted by MSCs are vital in controlling lung growth and inflammation (Zhao et al., 2021). Abundant in diverse bioactive substances like miRNAs, proteins, and lipids, extracellular vesicles can relay signals aiding in cell repair and regeneration (Huang et al., 2023). miRNAs delivered through extracellular vesicles have the capacity to control gene expression in target cells, thereby boosting their anti-inflammatory and reparative capacities (Charoenpong et al., 2023). Bone marrow stromal cells release extracellular vesicles containing miR-34c-5p, which mitigate lung damage and inflammation in bronchopulmonary dysplasia by targeting OTUD3 and promoting PTEN degradation (Wan et al., 2023).

**FIGURE 2:**
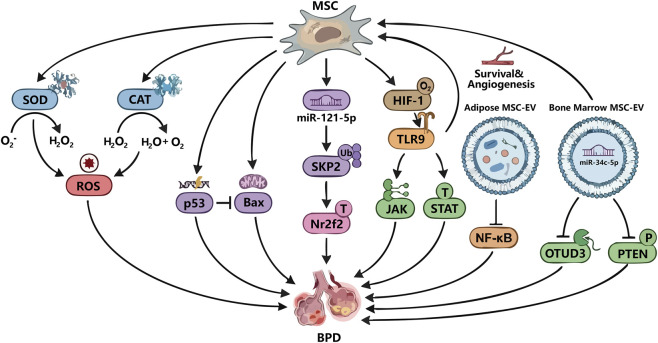
Studies have verified that MSCs mitigate oxidative harm by elevating the levels of SOD and CAT, thereby diminishing the levels of ROS within cells. MSCs are capable of increasing lung cell survival rates and aiding in healthy lung growth by obstructing apoptotic signaling routes like p53 and Bax pathways. By delivering miR-121-5p into lung cells, MSCs enhance its binding to SKP2, leading to a decrease in Nr2f2 ubiquitination and consequently mitigating lung damage caused by high oxygen. Transplanting mesenchymal stem cells through the HIF-1-TLR9 pathway enhances both their survival and angiogenic potential. Likewise, MSCs mitigate hyperoxia-induced multi-organ damage via the JAK/STAT pathway. Extracellular vesicles derived from adipose tissue MSCs mitigate hyperoxia-induced lung injury in neonatal rats by inhibiting the NF-κB signaling pathway. Studies have also shown that extracellular vesicles from bone marrow stromal cells with miR-34c-5p can mitigate lung damage and inflammation due to bronchopulmonary dysplasia by targeting OTUD3 to facilitate PTEN degradation.

The aforementioned studies indicate that MSCs significantly contribute to treating bronchopulmonary dysplasia (BPD) via various pathways such as immune regulation, lung tissue restoration, antioxidant properties, enhancement of lung function, and intercellular interaction. Not only do MSCs enhance pulmonary function in BPD sufferers, but they could also play a crucial role in averting the disease’s progression (Figure 2). Despite the promising outcomes of current studies, larger randomized controlled trials are still needed to confirm the long-term efficacy and safety of MSC-based therapy.

For BPD, most supportive data are derived from neonatal animal models and early-phase studies. Clinical translation is complicated by gestational age, postnatal oxygen exposure, infection, nutritional status, and the rapid developmental changes of the immature lung. Long-term safety is particularly important because treatment is administered during a critical window of lung and immune development. Follow-up should therefore include respiratory outcomes, growth, neurodevelopment, and immune-related adverse events.

### 2.3 The role and impact of MSCs on pulmonary arterial hypertension

Pulmonary arterial hypertension (PAH) remains a lethal disease, with no existing cure for this critical health issue (Liu H. et al., 2024). Post-diagnosis of PAH, patients typically live between 5 and 7 years (Jiang D. T. et al., 2022). Consequently, there is an urgent need to develop novel therapeutic strategies for PAH (Zhang et al., 2020). Increasing evidence points to stem cells emerging as an innovative and efficacious therapeutic approach, yet the comprehension of their underlying mechanisms remains restricted (Peeters et al., 2021). Research indicates that using MSCs has markedly enhanced physiological parameters associated with pulmonary arterial hypertension in animal models, encompassing pulmonary arterial pressure, cardiac output, and oxygenation (Tang et al., 2023). Initial clinical trials are underway to assess the safety and effectiveness of MSCs in treating PAH patients. The findings of this study suggest that MSC treatment improves symptoms and quality of life, underscoring its potential clinical applicability (Klinger et al., 2021). Studies indicate that MSCs from various origins, including bone marrow, adipose tissue, and umbilical cord blood, are highly effective in treating PAH, hinting at their extensive use as a therapeutic approach (Chi et al., 2024; Hu et al., 2022). Presently, it is widely accepted that MSCs influence the development of pulmonary arterial hypertension in PAH patients via mechanisms like compromised pulmonary endothelial function and excessive proliferation of pulmonary artery smooth muscle cells (Deng et al., 2024; Suwanmanee et al., 2023).

Studies indicate that MSCs are capable of encouraging the growth and movement of pulmonary endothelial cells, thereby boosting angiogenesis. Such capability is vital for improving pulmonary vascular circulation in individuals suffering from pulmonary arterial hypertension (Kuroyanagi et al., 2017; Tsiftsoglou, 2021). MSCs have the ability to lower the pressure in pulmonary arteries by encouraging the creation of new blood vessels. MSC treatment is known to improve endothelial function, enhance vasodilation, and consequently reduce pulmonary arterial pressure (Liu H. et al., 2024). By targeting HIF-1 and Runx2, exosomes derived from induced pluripotent stem cells reduce the remodeling of blood vessels in cases of pulmonary arterial hypertension (Liu P. et al., 2023). Extracellular vesicles derived from MSCs inhibit the development of pulmonary arterial hypertension via mechanisms dependent on microRNA-200^78^.

By secreting growth factors like VEGF and HGF, MSCs enhance the repair and regeneration of endothelial cells and smooth muscle cells in pulmonary arteries, thus bettering the structure and functionality of pulmonary blood vessels (Janowiak et al., 2022; Hou J. et al., 2023). MSCs are capable of safeguarding pulmonary artery smooth muscle cells and endothelial cells against oxidative damage and diminishing cell death via their antioxidant properties (Platenburg et al., 2023). By regulating HIF-1, Prostaglandin E1 lessens cell death and enhances the migration of MSCs in cases of pulmonary arterial hypertension (Zhang H. et al., 2024). EPO regulates the proliferation and migration of bone marrow MSCs via the Notch1/Jagged pathway in treating pulmonary arterial hypertension (Chakraborty et al., 2024). Modification MSCs with superoxide dismutase mitigates high-altitude pulmonary edema by suppressing oxidative stress via the Nrf2/HO-1 pathway (Sun et al., 2022).

**FIGURE 3:**
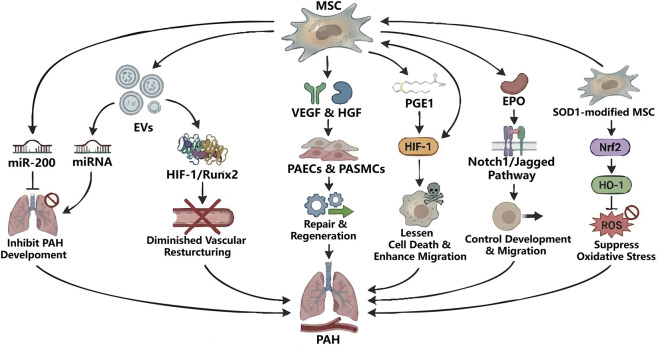
Studies indicate that extracellular vesicles derived from pluripotent stem cells reduce vascular remodeling in pulmonary arterial hypertension by targeting HIF-1 and Runx2. Additionally, these vesicles can inhibit the development of pulmonary arterial hypertension via mechanisms dependent on microRNA-200. By secreting VEGF and HGF, MSCs enhance the repair and regeneration of endothelial cells and smooth muscle cells in pulmonary arteries, leading to better structure and functionality of these blood vessels. By regulating HIF-1, Prostaglandin E1 lessens cell death and enhances the migration of mesenchymal stem cells in cases of pulmonary arterial hypertension. Through the Notch1/Jagged pathway, EPO plays a crucial role in regulating the proliferation and migration of bone marrow mesenchymal stem cells, aiding in the management of pulmonary artery hypertension. Altering mesenchymal stem cells with superoxide dismutase one mitigates high-altitude pulmonary edema by suppressing oxidative stress via the Nrf2/HO-1 pathway.

The aforementioned studies indicate that MSCs play a crucial role in managing pulmonary arterial hypertension by facilitating changes in pulmonary blood vessels, enhancing antioxidant capacity, and aiding in cell repair. MSCs improve pulmonary vascular function and may play a crucial role in slowing the progression of PAH (Figure 3).

In PAH, MSC-based therapy is biologically attractive because endothelial dysfunction, vascular inflammation, smooth muscle cell proliferation, and right ventricular remodeling are central to disease progression. However, available evidence is still mainly preclinical or early clinical. Cell retention in the pulmonary circulation, effects on vascular remodeling, arrhythmogenic or pro-thrombotic risks, and interactions with standard PAH therapies require systematic evaluation before routine clinical use.

### 2.4 The role and impact of MSCs on pulmonary fibrosis

The global incidence of Idiopathic pulmonary fibrosis (IPF) is increasing, yet the range of available treatments remains restricted (He et al., 2020; Lim et al., 2020; Sang et al., 2022). Currently, traditional therapies that depend on medication have not yielded optimal outcomes in counteracting lung damage or extending the lifespan of IPF patients (Chen et al., 2022). Recent advancs in stem cell therapy have highlighted the effectiveness of intravenous stem cell infusion in addressing critical pulmonary fibrosis (Atanasova et al., 2022). Early clinical trials have indicated the safety and potential enhancement of lung function metrics, life quality, and symptoms of pulmonary fibrosis in patients using MSCs (Nicolle, 1985; Bari et al., 2021). The findings endorse the use of MSCs as a approach for the treatment of pulmonary fibrosis (Court et al., 2020). Extended research indicates that MSC therapy could beneficially influence the long-term management of chronic pulmonary fibrosis patients, potentially slowing the disease’s advancement and enhancing life quality. Ongoing studies aim to validate the long-term efficacy of MSCs in this field (Bonifazi et al., 2020; Choi et al., 2023). Nonetheless, it encounters constraints like the host’s inability to regulate cell activity and swift elimination post-implantation. Earlier research has systematically summarized inflammation, immune imbalance, and extracellular matrix alterations within the IPF microenvironment, aiming to identify MSC-based approaches for treating IPF by targeting the lung microenvironment (Cassandras et al., 2020; Escarrer-Garau et al., 2024).

In this context, our primary attention is on the most recent advancements in research within this area. MSCs are capable of reducing lung inflammation through the release of anti-inflammatory cytokines like IL-10, TGF-β, and HGF. These factors are capable of obstructing the activation and infiltration of pro-inflammatory cells, thereby diminishing inflammation intensity in pulmonary tissue (Bao et al., 2024). Studies indicate that MSCs play a crucial role in diminishing inflammation and ameliorating histopathological alterations in models of pulmonary fibrosis (Han et al., 2023; Barrère-Lemaire et al., 2024). MSCs can enhance the anti-inflammatory response by promoting macrophage polarization toward the M2 phenotype. M2 macrophages can promote tissue repair and regeneration and may mitigate the progression of fibrosis (Li et al., 2021). Furthermore, MSCs have the ability to modulate T-cell function, suppress their inflammatory activity, and enhance the immune microenvironment (Li et al., 2022).

It has been demonstrated that MSCs enhance the proliferation and differentiation of alveolar epithelial cells and fibroblasts through the release of various growth factors, including VEGF, HGF, FGF, and more. Such growth factors are capable of promoting cellular regeneration and enhancing the reparative capacity of injured lung tissue (Li et al., 2022; Mercader-Barceló et al., 2023).

An overabundance of extracellular matrix accumulation primarily contributes to the advancement of pulmonary fibrosis. MSCs improve extracellular matrix homeostasis and reduce collagen accumulation by inhibiting excessive fibroblast proliferation and promoting the production of matrix metalloproteinases (Nataliya et al., 2023; Yuan et al., 2022).

Elevated oxidative stress frequently accompanies pulmonary fibrosis, and MSCs are capable of lowering the levels of intracellular ROS through the amplification of antioxidant enzymes like SOD and CAT. This process aids in reducing oxidative damage and safeguarding lung cells (Long et al., 2024; Liu Q. et al., 2023).

Early-stage research indicates that administration of MSCs markedly enhances both respiratory function and physiological parameters in mouse models of pulmonary fibrosis (Zhao et al., 2023). By restoring injured lung tissue, MSCs can improve pulmonary gas exchange and overall patient condition (Zhang X. et al., 2022; Hou L. et al., 2023). In models of pulmonary fibrosis, MSCs have been demonstrated to decrease both histological scores and collagen accumulation. This suggests that MSCs are capable of diminishing the extent of pulmonary fibrosis and enhancing the architecture of lung tissue (Zhang W. Y. et al., 2024; Warren et al., 2023).

In treating pulmonary fibrosis using MSCs, extracellular vesicles are vital, being essential for cellular interaction (Shi et al., 2022). Recent studies in this field primarily concentrate on the interaction of information among extracellular vesicles, MSCs, and epithelial cells during pulmonary fibrosis. Studies indicate that employing modified exosomes derived from MSCs is effective in targeting senescent alveolar epithelial cells to treat pulmonary fibrosis (Dhiman et al., 2021). Extracellular vesicular miR-17-5, derived from human embryonic stem cells, inhibits pulmonary fibrosis by targeting platelet-derived reactive protein-2 (Wu T. et al., 2023). miR-466f-3, carried by extracellular vesicles derived from MSCs, counteracts the EMT in radiation-triggered lung damage by obstructing the AKT/GSK3 pathway via c-MET (Yang et al., 2024). The miR-218 in extracellular vesicles, originating from MSCs, impedes the shift from endothelial to mesenchymal cells through the epigenetic regulation of BMP2 in lung fibrosis (Gao et al., 2024). Extracellular vesicles derived from MSCs and carrying microRNA-30 reduce the Runx1/Spred2 axis, thereby preventing pulmonary fibrosis (Jung et al., 2019). Extracellular vesicles derived from MSCs alleviate murine cytomegalovirus pneumonia via the NF-κB/NLRP3 signaling pathway. Extracellular vesicles derived from bone marrow MSCs inhibit the IL-33/ST2 pathway via microRNA-214, thereby reducing skin fibrosis in systemic sclerosis. (Jiang Y. J. et al., 2022). Wnt/β-catenin signaling plays a role in the repair of early-stage pulmonary fibrosis associated with acute respiratory distress syndrome via extracellular vesicles released by MSCs. (Nobre et al., 2022). It has been demonstrated that extracellular vesicles derived from MSCs can reduce lung inflammation and fibrosis in mice caused by silica via the miR-223-3p/NLRP3 axis (Liang et al., 2020). Modifications to S-RBD, originating from MSCs and exosomes engineered by miR-486-5, are effective in preventing ferroptosis and mitigating lung damage and prolonged pulmonary fibrosis caused by radiation (Liu et al., 2021).

**FIGURE 4:**
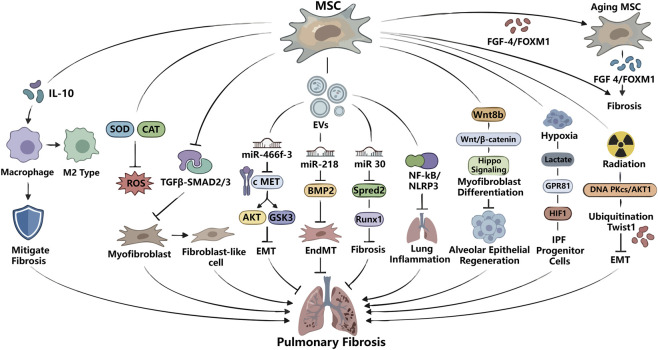
MSCs can release IL-10 to reduce lung inflammation, promote macrophage polarization toward the M2 phenotype, and mitigate fibrosis progression. By increasing SOD and CAT levels, MSCs can reduce intracellular ROS levels. Exosomal miR-466f-3 counteracts EMT by inhibiting the AKT/GSK3 pathway via c-MET. miR-218 inhibits the endothelial-to-mesenchymal transition through epigenetic regulation of BMP2 in pulmonary fibrosis. Extracellular vesicles carrying microRNA-30 reduce the Runx1/Spred2 axis, thereby preventing pulmonary fibrosis. Studies also verify that extracellular vesicles decelerate lung inflammation progression via the NF kappaB/NLRP3 signaling pathway. Recent studies have revealed that mesenchymal stem cells primarily play a part in pulmonary fibrosis through specific signaling pathways. The process of alveolar epithelial regeneration in pulmonary fibrosis is hindered by Hippo signaling. Hypoxia intensifies the fibrosis in IPF mesenchymal progenitor cells via the lactate/GPR81/HIF1 pathway. Mesenchymal stem cells residing in aged lungs are responsible for inducing pulmonary fibrosis via the FGF-4/FOXM1 axis. In radiation-induced pulmonary fibrosis, DNA-PKcs/AKT1 inhibits the epithelial-mesenchymal transition by promoting ubiquitination and degradation of Twist1. By inhibiting the TGF-β/SMAD pathway, mesenchymal stem cells can reversibly transform myofibroblasts into fibroblast-like cells. Wnt8b regulates myofibroblast differentiation in lung mesenchymal stem cells by activating the Wnt/β-catenin signaling pathway in pulmonary fibrosis.

Recent studies have revealed that MSCs primarily play a part in pulmonary fibrosis through specific signaling pathways. Hippo signaling inhibits the repair of alveolar epithelial cells in pulmonary fibrosis (Wu et al., 2020). Hypoxia exacerbates the fibrosis in IPF mesenchymal progenitor cells via the lactate/GPR81/HIF1 pathway (Chen S. et al., 2024). MSCs in aged lungs are responsible for inducing pulmonary fibrosis via the FGF-4/FOXM1 pathway (He et al., 2024). During radiation-induced pulmonary fibrosis, DNA-PKcs/AKT1 inhibits epithelial-mesenchymal transition by triggering ubiquitination and the degradation of Twist1. By inhibiting the TGF-β/SMAD2/3 pathway, MSCs can reversibly transform myofibroblasts into fibroblast-like cells. Wnt8b regulates myofibroblast differentiation in lung-resident MSCs by activating the Wnt/β-catenin pathway in pulmonary fibrosis (Bandeira et al., 2023). In summary, MSCs have demonstrated notable therapeutic effects in pulmonary fibrosis through multiple mechanisms, including immune regulation, tissue repair, antioxidant effects, and improvement of lung function (Figure 4).

In pulmonary fibrosis, the therapeutic application of MSCs requires caution. MSCs may suppress inflammation and attenuate extracellular matrix deposition in experimental models, but resident or disease-associated mesenchymal progenitor cells can also contribute to myofibroblast expansion and fibrotic remodeling. The efficacy window may be narrow, because established fibrosis is less reversible than early inflammatory injury. Future studies should distinguish anti-inflammatory effects from true antifibrotic activity, define optimal timing, and evaluate whether repeated dosing or engineered MSC-EVs can improve tissue targeting without increasing profibrotic risk.

### 2.5 The role and impact of MSCs on lung cancer

Lung cancer, known for its high occurrence rate, has emerged as a highly sought-after research area. Further comprehensive investigation is required into the interactions between MSCs and lung cancer cells.

Current studies indicate that MSCs have the ability to influence the tumor’s microenvironment and encourage the growth of tumor cells through the release of cytokines like TGF-β (Hawthorne et al., 2023). The stability of RMRP, driven by methylation, fosters the growth and advancement of non-small cell lung cancer through the control of the TGFBR1/SMAD2/SMAD3 pathway. Mesenchymal stem cell-derived extracellular vesicles have served as carriers for delivering non-coding RNAs in pulmonary disorders. It has been demonstrated that extracellular vesicles derived from bone marrow MSCs impede the growth of non-small cell lung cancer cells by inhibiting the PI3K/AKT pathway (Dong et al., 2021). Modified exosomes derived from bone marrow MSCs, carrying miRNAs, have been demonstrated to enhance the growth of non-small cell lung cancer (Lin et al., 2022).

Tumor angiogenesis is considered a crucial factor in tumor development. Research indicates that MSCs promote tumor angiogenesis by secreting angiogenic factors such as VEGF and bFGF. Angiogenesis serves a dual purpose: supplying essential nutrients and oxygen to tumor cells and facilitating their metastasis (Feng et al., 2022) MSCs increase the aggressive nature of lung cancer cells through their interaction with tumor cells. The primary mechanism for this improvement is the upregulation of genes like matrix metalloproteinases (MMPs) and urokinase-type plasminogen activator (uPA), which play a significant role in cell migration and invasion (Kim et al., 2022; Kun et al., 2024). The interaction between cells hastens tumor proliferation. Comparable research indicates that Fangqinoline (Fan) inhibits the metastasis of non-small cell lung cancer through the reversal of epithelial-mesenchymal transition and the inhibition of the Akt/mTOR signaling pathway in the cytoplasm. Exosomes derived from murine mesenchymal stem cells inhibit the AKT/GSK3 pathway, thereby counteracting EMT in radiation-induced lung injury (Wu et al., 2026). miR-145-5p, derived from bone marrow MSCs, inhibits the progression of non-small cell lung cancer cells by targeting SOX9. This study also revealed that cigarette smoke enhances osteopontin levels, recruits MSCs, and promotes the metastasis of lung cancer (Massoumi et al., 2026). ZFP281 initiates a dormancy process similar to mesenchymal in early-stage breast cancer cells, aiming to inhibit the metastatic growth of cancer cells in the lungs (Teo et al., 2025). Mesenchymal stem cell-derived extracellular vesicles also inhibit the proliferation and metastasis of non-small cell lung cancer by targeting THBS2 through miR-598 (Xu et al., 2026).

MSCs not only enhance the invasive capabilities of lung cancer cells but also contribute to the survival of these cells. Studies indicate that, through the release of factors such as HGF and IL-6, tumor cells can resist apoptosis and enhance their survival (Yang et al., 2026). As an illustration, HGF is capable of inhibiting the apoptotic signaling pathways in cancer cells, thereby enhancin drug resistance (Huang et al., 2026). Likewise, MSCs facilitate immune evasion by cancer cells by suppressing the functions of effector T cells and natural killer (NK) cells. For example, MSCs can secrete prostaglandin E2 (PGE2) and indoleamine 2,3-dioxygenase (IDO), thereby suppressing antitumor immune responses and helping tumor cells evade immune surveillance (Aziziyan et al., 2026; Mussin et al., 2025). It has been demonstrated that MSCs impact both NK cells and macrophages within immune cells. M2 macrophages commonly play a role in inhibiting the immune system and aiding in tissue healing, thereby aiding in tumor survival and growth (Che et al., 2026). MSCs have the capability to modify macrophage polarization states, facilitating their evolution into the pro-tumor M2 type. Such a change aids not just in the expansion of tumors but could also encourage their metastasis (Maruiwa et al., 2026; Zhang Yumeng et al., 2026).

Nonetheless, certain research indicates that MSCs may encourage tumor recurrence under specific circumstances. The reason lies in their function within the tumor’s microenvironment, potentially resulting in the endurance and proliferation of remaining tumor cells post-treatment (Shu et al., 2026). Consequently, this aspect must be thoroughly contemplated in clinical applications.

**FIGURE 5:**
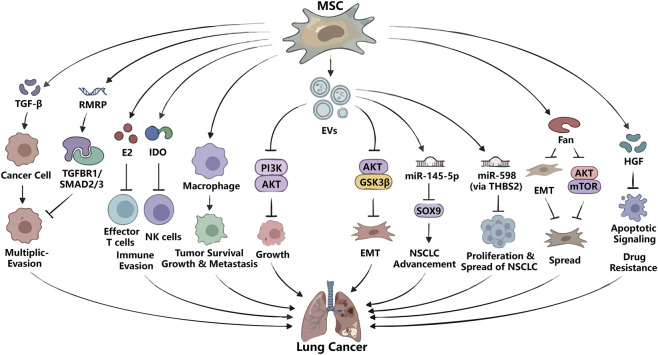
MSCs can secrete TGF-β, which promotes the proliferation of cancer cells. RMRP promotes the growth and progression of lung cancer through regulation of the TGFBR1/SMAD2/SMAD3 pathway. The growth of lung cancer cells is inhibited by extracellular vesicles, which obstruct the PI3K/AKT pathway. Fan inhibits lung cancer metastasis by reversing EMT and suppressing the Akt/mTOR pathway. Additionally, extracellular vesicles can inhibit the AKT/GSK3β pathway, thereby reversing EMT. By targeting SOX9, exosomal miR-145-5p inhibits the progression of non-small cell lung cancer cells. This research also discovered that extracellular vesicles originating from mesenchymal stem cells impede the proliferation and spread of non-small cell lung cancer by directing THBS2 to transmit miR-598. HGF can inhibit apoptotic signaling pathways in cancer cells, thereby enhancing drug resistance. Likewise, MSCs facilitate immune evasion by cancer cells by suppressing the functions of effector T cells and NK cells. MSCs are also capable of releasing E2 and IDO, which suppress immune reactions against tumors. It has been demonstrated that MSCs impact both NK cells and macrophages within immune cells. M2 macrophages commonly play a role in inhibiting the immune system and aiding in tissue healing, thereby aiding in tumor survival and growth. MSCs can modulate macrophage polarization, facilitating their transition toward the pro-tumor M2 phenotype. Such a change not only supports tumor growth but may also promote metastasis.

To sum up, MSCs play multifaceted roles in lung cancer, promoting tumor development, metastasis, and immune evasion, and could offer therapeutic possibilities in specific scenarios. Despite the varied functions of MSCs within the tumor’s microenvironment, their influence on tumors is contingent on distinct environmental elements and the types of tumors. Consequently, upcoming studies should explore the twofold function of MSCs in lung cancer to formulate better treatment approaches (Figure 5). For the therapeutic application of MSCs, it is essential to investigate their potential use in targeted and combination therapies while minimizing their tumor-promoting effects.

Lung cancer represents a distinct translational challenge because MSCs may exert both antitumor and tumor-supportive effects depending on tumor subtype, stromal context, and engineering strategy. Native MSCs can potentially promote angiogenesis, immune evasion, epithelial–mesenchymal transition, metastatic niche formation, or therapy resistance, whereas engineered MSCs or MSC-EVs may serve as targeted delivery vehicles for antitumor molecules. Therefore, clinical application in oncology should prioritize engineered and traceable products, tumor-specific safety assessment, biodistribution monitoring, and strict exclusion of pro-tumorigenic activity.

### 2.6 The role and impact of MSCs on asthma

Numerous research works have verified the advancements and future potential of MSCs in managing asthma (Li Zekun et al., 2026). By modifying MSCs, one can counteract the airway remodeling in persistent asthma (Li Jialin et al., 2026). Consistent with the mechanisms described above, MSCs have been shown to modulate asthma progression by affecting immune cells (Chen et al., 2026; Zhang Sha et al., 2026). MSCs are capable of facilitating the conversion of Th2 cells into Tregs and boosting the immune system’s resistance to allergic responses (Algariri et al., 2026). This process can reduce eosinophil infiltration, attenuate airway hyperresponsiveness, and alleviate the clinical symptoms of asthma (Lu et al., 2026). Typical alterations in the airways of asthma sufferers involve the thickening of airway walls and an overaccumulation of the extracellular matrix. MSCs ameliorate airway remodeling through the release of various growth factors, regulation of fibroblast activity, and reduction of collagen and other extracellular matrix components (Zhai et al., 2026). Comparable research has verified that miR-301, originating from adipose-derived MSCs, regulates airway smooth muscle cells in asthma cases by targeting STAT3 (Suzuki et al., 2026). Targeted inhibition of the Notch1/Jagged1 signaling pathway may improve the migration of bone marrow MSCs, thereby regulating asthma-associated immune inflammation. This role aids in reinstating the airway’s standard structure and functionality.

**FIGURE 6:**
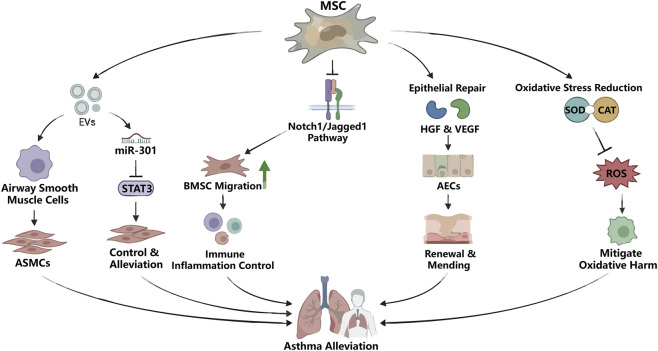
miR-301, carried by extracellular vesicles derived from adipose-derived mesenchymal stem cells, regulates airway smooth muscle cells in asthma by targeting STAT3. Targeted inhibition of the Notch1/Jagged1 signaling pathway may improve the migration of bone marrow mesenchymal stem cells, thereby regulating asthma-associated immune inflammation. MSCs are also capable of aiding in the renewal and mending of airway epithelial cells through the release of HGF and VEGF. MSCs mitigate airway oxidative damage by boosting the levels of antioxidant enzymes SOD and CAT, thereby lowering ROS concentrations.

MSCs are also capable of aiding in the regeneration and repair of airway epithelial cells through the release of substances like HGF and VEGF. Enhancing the function of the airway barrier and lowering allergic reaction rates is essential (Kim et al., 2022). In the pathogenesis of asthma, oxidative stress is a significant factor. MSCs mitigate airway oxidative damage by boosting the levels of antioxidant enzymes like SOD and CAT, thereby lowering ROS concentrations (Figure 6). This process aids in safeguarding airway cells and mitigating asthma symptoms (Fasae et al., 2026).

In asthma, MSCs and MSC-EVs may regulate Th2/Treg balance, eosinophilic inflammation, airway epithelial repair, oxidative stress, and airway remodeling. Nevertheless, asthma is a heterogeneous disease with allergic, eosinophilic, neutrophilic, and steroid-resistant phenotypes. Evidence from animal models may not fully predict therapeutic efficacy in patients. Future studies should define phenotype-specific indications, inhaled or systemic delivery strategies, repeated-dose safety, and clinically meaningful endpoints such as exacerbation frequency, lung function, airway hyperresponsiveness, and biomarker responses.

**FIGURE 7:**
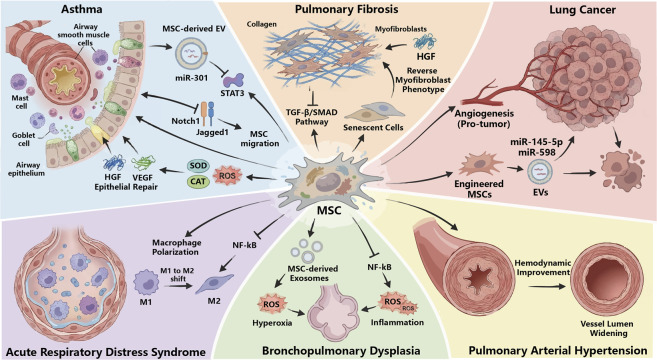
Mesenchymal stem cells exhibit multifaceted therapeutic properties in respiratory diseases through multiple mechanisms, including immune regulation, improvement of oxygenation, exosome-mediated communication, and antioxidant effects.

In summary, mesenchymal stem cells exhibit multifaceted therapeutic properties in respiratory diseases through multiple mechanisms, including immune regulation, improvement of oxygenation, exosome-mediated communication, and antioxidant effects. In certain microenvironments, however, these cells may also promote cellular proliferation, tumor progression, and immune evasion, while under specific circumstances they may still serve as potential therapeutic tools. The interplay of these processes renders MSCs an encouraging treatment approach, yet additional extensive clinical studies are required to confirm their sustained effectiveness and safety (Tables 1, 2) (Figure 7).

**TABLE 1:** Therapeutic roles and clinical translation status of MSCs in major respiratory diseases.

Disease	Role and evidence of MSCs	Clinical translation status	References
Acute Respiratory Distress Syndrome (ARDS)	Suppresses cytokine storm, reduces alveolar macrophage pyroptosis, promotes epithelial repair, and improves oxygenation	Supported by multiple animal studies; early-phase clinical trials show good safety, but efficacy requires validation in large-scale trials	Curley et al. (2024), Liang et al. (2023), Batchinsky et al. (2023), Morrison et al. (2017), Masterson et al. (2021), Lv et al. (2020), Millar et al. (2020)
Bronchopulmonary Dysplasia (BPD)	Anti-inflammatory, promotes alveolar and vascular development, antioxidant, and mitigates hyperoxia-induced injury	Effective in neonatal animal models; several Phase I/II clinical trials are ongoing	Cyr-Depauw et al. (2024), Moreira et al. (2020), Chaudhury et al. (2019), Chen et al. (2024b), Caplan, 2016; Xu et al. (2021), Yang et al. (2022)
Pulmonary Arterial Hypertension (PAH)	Promotes angiogenesis, improves endothelial function, inhibits abnormal smooth muscle proliferation, and reduces pulmonary arterial pressure	Significantly improves hemodynamics in animal models; initial human trials demonstrate symptom relief and improved quality of life	Zhao et al. (2021), Wan et al. (2023), Peeters et al. (2021), Tang et al. (2023), Klinger et al. (2021), Liu et al. (2023a)
Pulmonary Fibrosis (e.g., IPF)	Inhibits TGF-β/SMAD pathway, reverses myofibroblast phenotype, reduces collagen deposition, and targets senescent cells	Early clinical trials show trends toward improved lung function and quality of life; long-term efficacy remains to be confirmed	Ya et al. (2023), Janowiak et al. (2022), Hou et al. (2023a), Sang et al. (2022), Chen et al. (2022), Bonifazi et al. (2020), Choi et al. (2023), Mercader-Barceló et al. (2023), Nataliya et al. (2023), Zhang et al. (2024b)
Lung Cancer	Dual role: may promote tumor angiogenesis and immune evasion; however, engineered MSCs can deliver anti-tumor miRNAs (e.g., miR-145-5p, miR-598)	Therapeutic application is still exploratory; tumor-promoting risks require careful evaluation; potential as a drug delivery vehicle is promising	Wu et al. (2023b), Yang et al. (2024), Jiang et al. (2022b)
Asthma	Modulates Th2/Treg balance, inhibits airway remodeling, reduces eosinophil infiltration, and repairs airway epithelium	Effective in animal models; limited clinical studies suggest reduced airway hyperresponsiveness and inflammation	Hawthorne et al. (2023), Feng et al. (2022), Kun et al. (2024)

**TABLE 2:** Therapeutic roles and clinical translation status of MSC-derived extracellular vesicles in major respiratory diseases.

Disease/Condition	Intervention	Design/Status focus	References
ARDS	Allogeneic bone marrow-derived MSCs	Phase 2b, randomized, double-blind, placebo-controlled, multicenter; single IV dose	Cyr-Depauw et al. (2024), Moreira et al. (2020)
ARDS	Human umbilical cord-derived MSCs	Open-label, single-center, dose-escalation safety/efficacy study	Klinger and Matthay (2022)
BPD prevention/treatment	PNEUMOSTEM®; human umbilical cord blood-derived MSCs	Open-label, single-center, dose-escalation study in premature infants at high risk for BPD	Shokravi et al. (2022), Yang et al. (2022)
BPD risk in preterm neonates	Stem cell-derived extracellular vesicles (UNEX-42)	Safety study of intravenous EVs in preterm neonates at high risk for BPD	Zhao et al. (2021)
Extreme prematurity/BPD prevention	Mesenchymal stromal cell therapy	Clinical trial evaluating safety and efficacy in extreme preterm infants	Suwanmanee et al. (2023), Liu et al. (2023a)
Idiopathic pulmonary fibrosis	Human umbilical cord-derived MSCs	Phase I/IIa study evaluating safety and tolerability	Zhang et al. (2024a)
Idiopathic pulmonary fibrosis	Menstrual blood-derived MSCs	Clinical trial exploring MSC therapy for IPF	Chakraborty et al. (2024)
Chronic lung diseases including COPD/IPF/asthma/ARDS	MSC-derived extracellular vesicles	Clinical study of nebulized inhalation of MSC-derived EVs	Bao et al. (2024)
Asthma	IFN-γ-primed MSCs or MSC-based strategies	Clinical studies in moderate-to-severe persistent or chronic asthma	Mercader-Barceló et al. (2023), Nataliya et al. (2023)
Pulmonary fibrosis/sarcoidosis/pulmonary hypertension/ILD	Mesenchymal stem cells	Study evaluating MSCs in subjects with chronic pulmonary diseases	Wu et al. (2023b), Yang et al. (2024), Jiang et al. (2022b)

### 2.7 MSC-derived extracellular vesicles as cell-free therapeutics in respiratory failure

MSC-EVs have attracted increasing attention as a cell-free therapeutic strategy for respiratory failure and ARDS. Compared with the administration of whole MSCs, MSC-EVs may retain part of the paracrine activity of parent cells while avoiding several challenges associated with live-cell delivery, including variable cell survival, limited homing efficiency, pulmonary microvascular trapping, batch-to-batch heterogeneity, and theoretical risks related to uncontrolled engraftment or unwanted differentiation (Zhang Chuxin et al., 2026). MSC-EVs carry bioactive proteins, lipids, and nucleic acids that can modulate macrophage polarization, suppress excessive inflammatory signaling, stabilize epithelial and endothelial barriers, reduce oxidative stress, and promote tissue repair (Martinez-Zalbidea et al., 2026). These effects overlap with many mechanisms attributed to MSCs, supporting the concept that EVs may represent one of the major mediators of MSC paracrine activity.

However, MSC-EVs should not be regarded simply as interchangeable substitutes for MSCs. Whole MSCs are living products that can respond dynamically to inflammatory or hypoxic microenvironments, whereas EVs are acellular products with finite cargo content, limited biological persistence, and activity that depends strongly on parental cell source, culture conditions, isolation method, particle dose, storage conditions, and potency assays (Shah et al., 2026). For this reason, the term ‘MSC-derived extracellular vesicles’ is preferable unless the endosomal origin of vesicles has been rigorously demonstrated (Bonilla et al., 2026). Translational studies should report particle number, protein content, vesicle size distribution, EV markers, negative markers, sterility, storage conditions, and functional potency testing to improve comparability across trials (Ashley et al., 2026).

Clinical evidence for MSC-EVs in respiratory failure is still emerging. A prospective, multicenter, double-blind, randomized, placebo-controlled phase II trial of bone marrow MSC-derived EVs, ExoFlo, in severe or critical COVID-19-associated respiratory failure reported that the 15-mL dose was safe, with no treatment-related adverse events and exploratory signals supporting further evaluation (Xue et al., 2026). In parallel, registered trials are evaluating MSC-EV-based products for ARDS and other pulmonary conditions, indicating that this field is moving from preclinical studies toward controlled clinical translation (Ley et al., 2026). Nevertheless, current evidence remains insufficient to establish routine clinical use. Future trials should clarify optimal dose, route of administration, timing relative to disease stage, repeat dosing strategy, and long-term safety, and should directly compare MSC-EVs with whole MSCs where scientifically and ethically appropriate.

## 3 Discussion

Lately, MSCs have emerged as a major focus of interest in biomedical research. With advancing research, a fundamental link has been established between MSCs and the development of respiratory disorders. Large-scale data analyses have supported the involvement of MSCs in various respiratory disorders. Furthermore, recent studies indicate that MSCs influence respiratory diseases through several mechanisms, including immune regulation, anti-inflammatory effects, promotion of cell repair and regeneration, and inhibition of cell death. By analyzing extensive literature, it becomes apparent that extracellular vesicles derived from MSCs are vital in the pathogenesis and treatment of respiratory disorders.

Studies have shown that MSCs play important roles in the treatment of acute respiratory distress syndrome, pulmonary fibrosis, and asthma through multiple mechanisms, including immune regulation, tissue repair, improvement of oxygenation, exosome-mediated effects, and antioxidant activity. The interplay of these processes renders MSCs an promising therapeutic strategy, yet additional extensive clinical studies are required to confirm long-term efficacy and safety. Likewise, it has been demonstrated that MSCs play a crucial role in ameliorating bronchopulmonary dysplasia by offering antioxidant properties, enhancing lung function, and facilitating intercellular communication. MSCs may not only improve pulmonary function in bronchopulmonary dysplasia but also help prevent disease progression. Despite the promising outcomes of recent studies, there remains a need for more extensive randomized controlled trials to confirm its prolonged effectiveness and safety.

Research indicates that MSCs may help treat pulmonary arterial hypertension by modulating pulmonary vascular remodeling, enhancing antioxidant capacity, and promoting cellular repair. Not only do MSCs enhance the physiological performance of pulmonary arteries, but they might also be crucial in hindering the advancement of pulmonary arterial hypertension. In contrast, MSCs play multifaceted roles in lung cancer, fostering tumor development, metastasis, and immune evasion, and could offer therapeutic possibilities in specific scenarios.

Despite the varied functions of MSCs within the tumor’s microenvironment, their influence on tumors varies based on distinct environmental elements and types of tumors. Consequently, upcoming studies should explore the twofold function of MSCs in lung cancer to formulate more efficacious treatment approaches. Investigating the potential use of MSCs in both targeted and combination therapies is crucial for therapeutic approaches, especially in reducing their tumor-promoting effects.

Recently, this domain has achieved satisfying advancements, thanks to the ongoing enhancement of associated technological methods. Recent years have seen a surge in research indicating the utility of iPSC-derived lung cancer organoid models in assessing extracellular vesicles derived from iPSC-derived mesenchymal stromal cells. This approach will enhance the methods for assessing the function of MSCs within the respiratory system. Nanomaterials, as a representative example of the integration of medicine and engineering, have also shown promising results in this field. Research validated the suppressive impact of nanoscale lysates derived from diverse dental pulp stem cells within a lung cancer model.

Likewise, engineered MSCs, similar to enzymes and nanoparticles, emit HGF to enhance the visual treatment of idiopathic pulmonary fibrosis in living organisms. It is our belief that as medicine and engineering increasingly merge, the scope of this domain will expand further in the times ahead.

Despite substantial progress, several obstacles continue to limit the clinical translation of MSC-based therapies for respiratory diseases. Differences in cell source, donor background, culture conditions, passage number, administration route, and outcome assessment make it difficult to compare results across studies. In addition, many available data are derived from animal models, whereas controlled clinical evidence remains limited for several disease areas. Whole-cell therapy also faces biological and logistical challenges, including short *in vivo* persistence, limited tissue homing, pulmonary trapping after intravenous infusion, and variable responses to host inflammatory microenvironments.

These limitations provide a rationale for developing MSC-EVs as cell-free therapeutic products. MSC-EVs may preserve important paracrine effects of MSCs while avoiding some problems associated with viable cell administration. Because EVs are acellular, they may be easier to standardize, store, dose, and engineer than whole MSCs. Their small size and cargo-carrying capacity also make them attractive delivery vehicles for anti-inflammatory, barrier-protective, and pro-repair signals in respiratory failure. However, MSC-EVs also introduce new challenges. Their biological activity depends on parental cell identity, preconditioning method, isolation strategy, particle purity, cargo composition, and storage conditions. Therefore, future studies should not only test whether MSC-EVs are effective, but also define potency assays that link EV characteristics to clinically meaningful outcomes.

At present, the clinical translation of MSCs and MSC-EVs remains at an early stage. This situation represents a major translational challenge. Although MSC-EVs are important mediators of intercellular communication, MSC secretomes contain multiple bioactive components in addition to EVs, including soluble cytokines, growth factors, chemokines, metabolites, and extracellular matrix-associated factors. Future studies should therefore clarify the relative contribution of EV-dependent and EV-independent mechanisms by using standardized EV depletion, rescue experiments, cargo profiling, and functional potency testing. Such work will help determine whether MSC-EVs should be developed as stand-alone therapies, engineered delivery vehicles, or adjunctive products to complement conventional treatment strategies.

In summary, mesenchymal stem cells have demonstrated a “multi effect” therapeutic potential in various respiratory system diseases through immune regulation, tissue repair, antioxidant, and extracellular vesicle mediated signaling. Although most of the evidence remains at the preclinical or early clinical stage, this novel cell-based therapeutic strategy may provide new hope for patients with severe lung disease who respond poorly to conventional therapies. Further optimization of delivery strategies and clarification of long-term safety and efficacy will be needed (Liu et al., 2022; Qin et al., 2023; Turner et al., 2024; Zhuang et al., 2023; Guo et al., 2022).
